# Oligo(Lactic Acid)_8_-Docetaxel Prodrug-Loaded PEG-*b*-PLA Micelles for Prostate Cancer

**DOI:** 10.3390/nano11102745

**Published:** 2021-10-17

**Authors:** Lauren Repp, Christopher J. Unterberger, Zhengqing Ye, John B. Feltenberger, Steven M. Swanson, Paul C. Marker, Glen S. Kwon

**Affiliations:** 1 Pharmaceutical Sciences Division, School of Pharmacy, University of Wisconsin-Madison, Madison, WI 53705, USA; lrepp@wisc.edu (L.R.); cunterberger@wisc.edu (C.J.U.); steve.swanson@wisc.edu (S.M.S.); paul.marker@wisc.edu (P.C.M.); 2 Medicinal Chemistry Center, School of Pharmacy, University of Wisconsin-Madison, Madison, WI 53705, USA; zhengqing.ye@wisc.edu (Z.Y.); john.feltenberger@wisc.edu (J.B.F.)

**Keywords:** docetaxel, prodrug, polymeric micelle, prostate cancer

## Abstract

Docetaxel (DTX) is among the most frequently prescribed chemotherapy drugs and has recently been shown to extend survival in advanced prostate cancer patients. However, the poor water solubility of DTX prevents full exploitation of this potent anticancer drug. The current marketed formulation, Taxotere^®^, contains a toxic co-solvent that induces adverse reactions following intravenous injection. Nano-sized polymeric micelles have been proposed to create safer, water-soluble carriers for DTX, but many have failed to reach the clinic due to poor carrier stability in vivo. In this study, we aimed to improve micelle stability by synthesizing an ester prodrug of DTX, oligo(lactic acid)_8_-docetaxel (o(LA)_8_-DTX), for augmented compatibility with the core of poly(ethylene glycol)-*b*-poly(lactic acid) (PEG-*b*-PLA) micelles. Due to the enhancement of drug-carrier compatibility, we were able to load 50% (*w*/*w*) prodrug within the micelle, solubilize 20 mg/mL o(LA)_8_-DTX (~12 mg/mL DTX-equivalent) in aqueous media, and delay payload release. While the micelle core prohibited premature degradation, o(LA)_8_-DTX was rapidly converted to parent drug DTX through intramolecular backbiting (t_1/2_ = 6.3 h) or esterase-mediated degradation (t_1/2_ = 2.5 h) following release. Most importantly, o(LA)_8_-DTX micelles proved to be as efficacious but less toxic than Taxotere^®^ in a preclinical mouse model of prostate cancer.

## 1. Introduction

Cancer remains a challenging disease to treat, with an estimated 19 million new cases throughout the world in 2020 [1]. Although much progress has been made in developing alternative treatment options, chemotherapy continues to be an effective tool in the fight against cancer. The taxanes in particular represent an important class of anticancer drugs that are frequently prescribed to treat a wide variety of solid tumors [2]. Docetaxel (DTX) is a potent taxane that binds to microtubules with high affinity to promote cell cycle arrest and subsequent apoptosis and is approved to treat cancers including breast cancer, non-small cell lung cancer, and castration-resistant prostate cancer [3,4]. Like many other chemotherapeutics, it is poorly water-soluble, requiring a mixture of Tween 80 and ethanol to effectively solubilize the drug for intravenous (IV) injection [5]. Tween 80 present in the marketed formulation Taxotere^®^, has been known to induce serious side effects and off-target toxicities, most notably causing hypersensitivity reactions and vesicular degeneration [6,7].

To address these solubility and formulation issues, researchers have utilized nanotechnology to create water-soluble carriers for small hydrophobic molecules for injection [8,9]. Composed of amphiphilic block copolymers, polymeric micelles can self-assemble in aqueous environments to encapsulate hydrophobic molecules within their hydrophobic core while providing water solubility through their hydrophilic shell [10]. The larger size of micelles (10–200 nm), relative to free drug molecules, prevents their extravasation into healthy tissue while taking advantage of the leaky vasculature of solid tumors through the enhanced permeability and retention (EPR) effect [11,12]. Recent nano-sized formulations of DTX have shown promise in preclinical cancer models [13,14,15,16,17,18,19,20], and a phase I clinical trial of a core cross-linked micellar DTX formulation was recently completed [21].

Clinical translation of these nanomedicines can be challenging, however, as many of these block copolymers function mainly as solubilizers rather than true nanocarriers. A number of factors, namely poor drug-carrier compatibility and the presence of blood proteins, contribute to premature degradation of the carrier and low drug retention in vivo [22]. Moreover, low drug loading necessitates high injection volumes. Chemical modification of drugs to create prodrugs for enhanced solubility and stability within polymeric matrices has been proposed to overcome these limitations [23,24]. Following release from the micelle, the promiscuity of esterases can be harnessed to cleave the promoiety to yield the bioactive parent drug for cancer cell death. Recently, our group has developed an oligo(lactic acid)_n_ (o(LA)_n_) ester prodrug of paclitaxel (PTX) for augmented stability in poly(ethylene glycol)-*block*-poly(lactic acid) (PEG-*b*-PLA) micelles. Addition of the o(LA)_n_ promoiety led to higher stability in blood, and o(LA)_8_-PTX-loaded micelles displayed an increased plasma area under the curve (AUC) and lower volume of distribution relative to PTX-loaded micelles. As a result of the extended drug exposure, greater antitumor efficacy was achieved in both 4T1 breast and A549 lung tumor models in mice [25].

**Figure 1:**
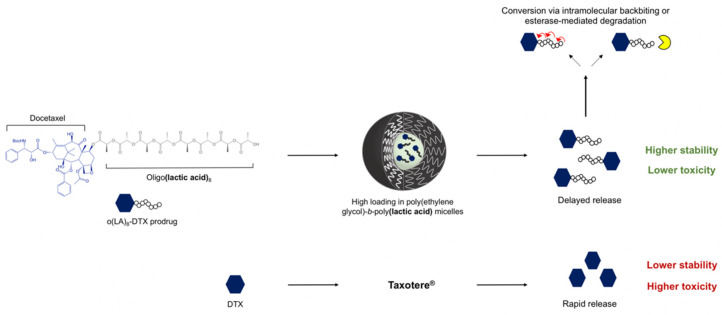
Improved formulation properties with o(LA)_8_-DTX vs. Taxotere^®^. Conjugation of the o(LA)_8_ promoiety leads to enhanced compatibility with the hydrophobic core of PEG-*b*-PLA micelles and greater payload stability. Following release, o(LA)_8_-DTX prodrug is converted to DTX through intramolecular backbiting or cleavage via plasma esterases.

Given the success of the PTX prodrug, the goal of this study was to create a stable, less toxic DTX formulation relative to the clinically prescribed Taxotere^®^ for prostate cancer treatment. We hypothesized that dose escalation was possible by removing the Tween 80 and ethanol required for drug solubilization. In order to circumvent the need for co-solvents, we synthesized an oligo(lactic acid)_8_-docetaxel prodrug, o(LA)_8_-DTX, for physical encapsulation in PEG-*b*-PLA micelles (Figure 1). Particle size, encapsulation efficiency, weight loading, stability, and release of o(LA)_8_-DTX-loaded micelles were characterized relative to parent drug DTX-loaded micelles. The in vitro conversion of o(LA)_8_-DTX prodrug was assessed in both aqueous media and plasma. Finally, the antitumor efficacy of o(LA)_8_-DTX micelles versus Taxotere^®^ was determined in nude mice bearing kidney capsule-grafted prostate cancer xenograft tumors. 

## 2. Materials and Methods

### 2.1. Materials

Docetaxel was purchased from LC Laboratories (Woburn, MA, USA). Tert-butyldimethylsilyl-protected oligo(lactic acid)_8_ (TBS-o(LA)_8_) was purchased from Proactive Molecular Research (Alachua, FL, USA). Poly(ethylene glycol)-*b*-poly(lactic acid) (PEG-*b*-PLA) copolymer was purchased from JenKem USA (Plano, TX, USA); with average M_n_ of PEG and PLA of 4000 and 2200 g/mol, respectively. Heparanized Sprague-Dawley rat and pooled human plasma were purchased from Innovative Research (Novi, MI, USA). Cancer cell lines were purchased from ATCC (Manassas, VA, USA). RPMI 1640 with L-glutamine was purchased from GenDEPOT (Katy, TX, USA). Fetal bovine serum (FBS) was purchased from Corning (Corning, NY, USA). Penicillin/streptomycin was purchased from Lonza (Basel, Switzerland). Ki67 primary antibody was purchased from Abcam (Cambridge, UK). Cleaved caspase-3 primary antibody was purchased from Cell Signaling Technology (Danvers, MA, USA). Citrate-based Unmasking Solution, biotinylated goat anti-rabbit IgG secondary antibody, Vectastain ABC Elite kit, and DAB Peroxidase Substrate Kit were purchased from Vector (Burlingame, CA, USA). CitriSolv was purchased from Deacon Labs (King of Prussia, PA, USA). Permount mounting medium was purchased from Fisher Scientific (Hampton, NH, USA). All other chemicals were purchased from Sigma Aldrich (St. Louis, MO, USA). Organic solvents were analytical grade and purchased from Fisher Scientific (Waltham, MA, USA).

### 2.2. Synthesis, Purification, and Characterization of Oligo(Lactic Acid)_8_-Docetaxel Prodrug (o(LA)_8_-DTX)

Proton nuclear magnetic resonance (^1^H NMR) data were collected on a Varian Unity-Inova three-channel 500 MHz NMR spectrometer (Varian Inc.; Palo Alto, CA, USA) in the Analytical Instrumentation Center within the School of Pharmacy, University of Wisconsin-Madison. The temperature was set to 25 °C and chemical shifts (δ) were reported in parts per million (ppm) relative to residual protonated chloroform-d (CDCl_3_) resonance at 7.26 ppm. All NMR spectra are included in the Supplementary Materials. 

Molecular mass and purity of the prodrug were confirmed with liquid chromatography-mass spectrometry (LCMS) using an Agilent 1290 Infinity II equipped with a 6120 Single Quadrupole MS (Agilent Technologies; Santa Clara, CA, USA). A gradient method was employed consisting of 5% methanol (MeOH) in H_2_O with 0.1% formic acid (mobile phase A) and MeOH with 0.1% formic acid (mobile phase B).

Tert-butyldimethylsilyl chloride (TBSCl) (696 mg, 4.62 mmol, 2.5 equiv) and imidazole (Im) (377 mg, 5.55 mmol, 3 equiv) were added to a solution of DTX (1.5 g, 1.85 mmol) in dimethylformamide (DMF) (3 mL) at 0 °C. The reaction mixture was slowly warmed to room temperature and stirred overnight. Ethyl acetate (EtOAc) (50 mL) was poured into the reaction mixture followed by washing with H_2_O (1 × 50 mL) and saturated NH_4_Cl (1 × 50 mL) solution. The organic layer was then collected, dried over MgSO_4,_ and filtered. Solvent was removed under reduced pressure and the resulting concentrate was purified via a CombiFlash Rf 4x system (Teledyne Isco Inc.; Lincoln, NE, USA) using a gradient elution of hexane and ethyl acetate (EtOAc). The residue was briefly dried in vacuo to yield 1.5 g of 2′-TBS-DTX (Figure S1). ^1^H NMR (400 MHz, Chloroform-*d*) δ 8.23 (d, *J* = 7.7 Hz, 2H), 8.08 (s, 1H), 7.71 (t, *J* = 7.4 Hz, 1H), 7.60 (t, *J* = 7.7 Hz, 2H), 7.49 (t, *J* = 7.4 Hz, 2H), 7.44–7.35 (m, 3H), 6.46–6.37 (m, 1H), 5.81 (d, *J* = 7.1 Hz, 1H), 5.69 (d, *J* = 8.9 Hz, 1H), 5.42 (d, *J* = 5.9 Hz, 1H), 5.35 (s, 1H), 5.11 (dd, *J* = 9.7, 2.3 Hz, 1H), 4.64 (s, 1H), 4.45 (d, *J* = 8.5 Hz, 1H), 4.42–4.31 (m, 3H), 4.17 (s, 1H), 4.05 (d, *J* = 7.1 Hz, 2H), 3.57 (s, 7H), 2.76–2.68 (m, 1H), 2.67 (s, 3H), 2.49 (d, *J* = 14.7 Hz, 1H), 2.03 (s, 3H), 2.02–1.94 (m, 1H), 1.86 (s, 3H), 1.43 (s, 8H), 1.37 (s, 4H), 1.24 (s, 3H), 0.86 (s, 8H), −0.18 (s, 2H).

N,N′-dicyclohexylcarbodiimide (DCC) (332 mg, 1.61 mmol, 1.5 equiv) and 4-dimethylaminopyridine (DMAP) (26 mg, 0.216 mmol, 0.2 equiv) were added to a solution of TBS-o(LA)_8_ (914 mg, 1.29 mmol, 1.2 equiv) in dichloromethane (DCM) and stirred for 1 h. Then 2′-TBS-DTX (1.0 g, 1.08 mmol) was added to the mixture and stirred at room temperature overnight. The resulting mixture was filtered and washed with H_2_O (1 × 30 mL) and saturated NaHCO_3_ (1 × 30 mL) solution. The organic layer was then collected, dried over MgSO_4,_ and filtered. Solvent was removed under reduced pressure and the resulting concentrate was purified with CombiFlash (Hexane/EtOAc). The residue was briefly dried in vacuo to yield 1.6 g of 2′-TBS-DTX-o(LA)_8_-TBS (Figure S2). ^1^H NMR (500 MHz, Chloroform-*d*) δ 8.43 (d, *J* = 7.7 Hz, 2H), 7.91 (t, *J* = 7.4 Hz, 2H), 7.81 (t, *J* = 7.7 Hz, 2H), 7.70 (t, *J* = 7.5 Hz, 2H), 7.64–7.55 (m, 4H), 6.61 (s, 1H), 6.00 (d, *J* = 7.1 Hz, 1H), 5.74 (d, *J* = 9.1 Hz, 1H), 5.69–5.60 (m, 2H), 5.56–5.41 (m, 5H), 5.32–5.26 (m, 1H), 4.86–4.82 (m, 1H), 4.71 (q, *J* = 6.7 Hz, 2H), 4.64 (d, *J* = 8.6 Hz, 1H), 4.51 (d, *J* = 8.5 Hz, 1H), 4.44 (q, *J* = 7.1 Hz, 2H), 4.33 (d, *J* = 7.8 Hz, 1H), 4.11 (d, *J* = 7.0 Hz, 1H), 3.80 (dd, *J* = 6.9, 4.0 Hz, 2H), 2.93–2.84 (m, 3H), 2.69 (dd, *J* = 15.4, 9.5 Hz, 2H), 2.65 (d, *J* = 4.3 Hz, 1H), 2.55–2.47 (m, 2H), 2.36 (s, 2H), 2.23 (d, *J* = 36.0 Hz, 5H), 2.00 (d, *J* = 3.3 Hz, 3H), 1.96 (d, *J* = 7.0 Hz, 3H), 1.94–1.83 (m, 12H), 1.76 (d, *J* = 6.7 Hz, 3H), 1.70–1.64 (m, 2H), 1.61 (s, 4H), 1.58 (d, *J* = 8.0 Hz, 2H), 1.56 (s, 2H), 1.45 (s, 2H), 1.40 (td, *J* = 12.0, 11.2, 3.5 Hz, 2H), 1.22 (s, 4H), 1.06 (s, 4H), 0.41 (d, *J* = 11.8 Hz, 4H), 0.32 (s, 2H), 0.20 (s, 2H), −1.63 (s, 1H).

Hydrogen fluoride pyridine (HF.Py) (1.0 M in THF, 1.5 mL, 1.5 mmol, 6 equiv) was added to a solution of 2′-TBS-DTX-o(LA)_8_-TBS (400 mg, 0.25 mmol) in tetrahydrofuran (THF) and stirred at room temperature for 4 h. The mixture was quenched with aq. NaHCO_3_ and extracted with EtOAc (3 × 15 mL). The crude product was purified with CombiFlash (Hexane/EtOAc). The residue was briefly dried in vacuo to yield 185 mg of o(LA)_8_-DTX (Figure S3). ^1^H NMR (500 MHz, CDCl_3_-d) δ 8.10 (d, J = 7.8 Hz, 2H), 7.62 (t, J = 7.4 Hz, 1H), 7.50 (t, J = 7.7 Hz, 2H), 7.39 (h, J = 6.2 Hz, 4H), 7.33 (td, J = 6.5, 2.8 Hz, 1H), 6.20 (s, 1H), 5.66 (d, J = 6.9 Hz, 1H), 5.51 (dd, J = 10.7, 7.2 Hz, 1H), 5.42 (d, J = 9.5 Hz, 1H), 5.32–5.24 (m, 1H), 5.26–5.13 (m, 7H), 4.96 (q, J = 7.0 Hz, 1H), 4.92 (dd, J = 9.4, 2.0 Hz, 1H), 4.62 (d, J = 5.0 Hz, 1H), 4.40–4.30 (m, 2H), 4.19 (d, J = 8.6 Hz, 1H), 3.98 (d, J = 6.8 Hz, 1H), 3.92 (d, J = 1.7 Hz, 1H), 3.48 (s, 1H), 3.33 (d, J = 5.4 Hz, 1H), 2.69 (d, J = 6.0 Hz, 1H), 2.54 (ddd, J = 14.5, 9.5, 7.3 Hz, 1H), 2.38 (s, 3H), 2.28 (d, J = 9.1 Hz, 2H), 2.02–1.89 (m, 1H), 1.88 (s, 3H), 1.66 (s, 1H), 1.64 (s, 2H), 1.64–1.54 (m, 19H), 1.47 (dd, J = 22.6, 7.0 Hz, 6H), 1.35 (s, 9H), 1.29–1.24 (m, 1H), 1.23 (s, 3H), 1.09 (s, 3H), 0.84 (t, J = 5.8 Hz, 1H), 0.75 (s, 0H).

### 2.3. Preparation and Characterization of o(LA)_8_-DTX-Loaded PEG_4k_-b-PLA_2.2k_ Micelles

PEG-*b*-PLA micelles containing DTX or o(LA)_8_-DTX were prepared using a thin film hydration method. Briefly, 1 mg of DTX or o(LA)_8_-DTX and 9 mg of PEG-*b*-PLA (for 10% wt loading) or 10 mg of o(LA)_8_-DTX and 10 mg of PEG-*b*-PLA (for 50% wt loading) were dissolved in 1 mL of acetonitrile (ACN) and transferred to a round-bottom flask. ACN was removed by reduced pressure using a rotary evaporator (Yamato Scientific; Santa Clara, CA, USA) at 60 °C to create a film. The film was subsequently dissolved at 60 °C using 1 mL of sterile water or saline. The solution was then filtered (0.22 μm, regenerated cellulose) to remove unencapsulated drug. Particle size was assessed using dynamic light scattering (DLS) on a Zetasizer Nano-ZS (Malvern Instruments; Worcestershire, UK) at 25 °C with a 173° measurement angle. The micelle solution was diluted in water to obtain a PEG-*b*-PLA concentration of ~0.1 mg/mL and the cumulants analysis was used to determine the z-average diameter and polydispersity index (PDI) of the micelles. Encapsulation efficiency and weight loading content were measured with reverse-phase high performance liquid chromatography (RP-HPLC) using a Shimadzu Prominence HPLC system (Shimadzu; Kyoto, Japan). The instrument was equipped with a LC-20AT pump, SIL-20AC HT autosampler, CTO-20AC column oven, and SPD-M20A diode array detector. DTX or o(LA)_8_-DTX were quantified with an Zorbax RX-C8 column (5 μm, 4.6 × 250 mm) (Agilent Technologies; Santa Clara, CA, USA) and isocratic method consisting of 70% ACN and 30% milliQ water with 0.1% methanol. Column temperature was set to 40 °C, flow rate was 1.0 mL/min, and the detection wavelength was 227 nm. Encapsulation efficiency was determined by dividing the post-filter drug level by the pre-filter drug level, and weight loading was determined by dividing the post-filter drug level by the sum of the drug and copolymer levels. Both particle size and drug content were monitored over time at 25 °C to assess stability of o(LA)_8_-DTX micelles. 

### 2.4. In Vitro Drug or Prodrug Release from Micelles

The release of drug or prodrug from PEG-*b*-PLA micelles was monitored in 10 mM phosphate buffered saline (PBS) at pH 7.4 under sink conditions. After loading DTX or o(LA)_8_-DTX into micelles, the micelle solutions were diluted to 0.5 mg/mL drug or prodrug content with 10 mM PBS (pH 7.4) and loaded into 20K molecular weight cut-off (MWCO) Slide-A-Lyzer dialysis cassettes (Thermo Scientific; Waltham, MA, USA). Dialysis cassettes were then placed in a 2 L PBS solution on a stirring hotplate at 37 °C. At predetermined time points, 100 μL of sample was withdrawn and replaced with 100 μL of PBS. The sample was then diluted with 900 μL of ACN and analyzed by RP-HPLC. Cumulative release over time was plotted and fitted using a one-phase exponential growth curve with GraphPad Prism 9.2.0 (San Diego, CA, USA).

### 2.5. Backbiting Conversion of o(LA)_8_-DTX

The intramolecular backbiting of o(LA)_8_-DTX prodrug was monitored at pH 7.4 (10 mM PBS) at 37 °C. First, o(LA)_8_-DTX was dissolved in ACN at 2 mg/mL, then diluted to 1 mg/mL in PBS. Microtubes containing 1 mL of this solution were placed in a 37 °C water bath under gentle shaking. At each time point, 50 μL of sample was withdrawn and diluted with 950 μL of ACN for RP-HPLC analysis. Additionally, backbiting of o(LA)_8_-DTX in micelles (10% and 50% loading) was assessed. Micelle solutions were diluted to 1 mg/mL in PBS and samples were evaluated using the same methods as with free o(LA)_8_-DTX. Curves were fit using a one-phase exponential decay model in GraphPad Prism 9.2.0 (San Diego, CA, USA).

### 2.6. In Vitro Plasma Stability of o(LA)_8_-DTX

The in vitro stability of o(LA)_8_-DTX in plasma was assessed in heparinized Sprague-Dawley rat plasma and pooled human plasma. Stock solution of o(LA)_8_-DTX in dimethyl sulfoxide (DMSO) was prepared at 5 mM, then diluted to 50 μM in plasma. Samples were placed in a 37 °C water bath with gentle shaking. At each time point, 100 μL of solution was removed and combined with 200 μL of ACN with 0.1% formic acid. Proteins were precipitated by centrifuging samples at 13,000 rpm for 10 min. The supernatant was then analyzed by RP-HPLC using a gradient method differing from the previously described isocratic method. The mobile phase consisted of ACN (A) and milliQ water with 0.1% methanol (B), and the gradient conditions were as follows: 0 min-50% A, 50% B; 22 min-80% A, 20% B; 24 min-50% A, 50% B. The flow rate was 1 mL/min, column temperature was 40 °C, and detection was at 227 nm. The amount of o(LA)_8_-DTX and its metabolites were calculated as a percentage of the starting level of o(LA)_8_-DTX, and the half-life was determined with GraphPad Prism 9.2.0 (San Diego, CA, USA). 

### 2.7. In Vitro Cytotoxicity of o(LA)_8_-DTX

The cell lines used for this study were authenticated by Short Tandem Repeat (STR) analysis performed by the University of Wisconsin Translational Research Initiatives in Pathology (TRIP) laboratory, and both PC3 (21 allelic polymorphisms across the 15 STR loci analyzed) and LNCaP (27 allelic polymorphisms across the 15 STR loci analyzed) cells were confirmed to match the known STR profiles for PC3 and LNCaP cells respectively with no mis-matched polymorphisms, indicating both were not contaminated with any other human cells. PC3 and LNCaP cells were maintained in media containing 10% (*v*/*v*) FBS and 1% (*v*/*v*) penicillin/streptomycin in RPMI-1640 base media with L-glutamine. Cell viability in culture was assessed using the Promega CellTiter-Blue Cell Viability Assay (Madison, WI, USA). Specifically, cells were plated at 400 cells/well in a 96-well plate 24 h before the addition of vehicle-, DTX- or o(LA)_8_-DTX-treated media. 48 h after treatment, 20 µL of CellTiter-Blue reagent was added to each well and fluorescence signal was read on a Fluostar Omega fluorescence plate reader (BMG Labtech; Ortenberg, Germany) at 544/590 nm.

### 2.8. In Vivo Antitumor Efficacy in Prostate Cancer Xenograft Model

Mice were housed in polysulfone cages containing corn cob bedding and maintained on a 12 h light and dark cycle at 20.5 ± 5 °C and 30–70% relative humidity. Mice were fed a 5015 Diet (PMI Nutrition International; Brentwood, MO, USA) from conception through weaning (postnatal day 21) and an 8604 Teklad Rodent Diet thereafter (Harlan Laboratories; Madison, WI, USA). Feed and water were available ad libitum. All procedures were approved by the University of Wisconsin Animal Care and Use Committee (M005521-R01-A03) and conducted in accordance with the NIH Guide for the Care and Use of Laboratory Animals. All animals were sacrificed by CO_2_ asphyxiation in accordance with guidelines set forth by the American Medical Veterinary Association. 

PC3 or LNCaP cells (3.5 × 10^5^) were combined with collagen mix containing 6 mg/mL rat tail collagen, PBS, 1 N NaOH, and distilled H_2_O to make 25 μL collagen pellets for xenograft surgery. Pellets were surgically grafted under the kidney capsules of 10-week-old male Balb/C nu/nu mice (Charles River; Wilmington, MA, USA). Each mouse had 2 xenografts: PC3 cells on one kidney and LNCaP cells on the other. Xenografts were grown in vivo for 5 weeks at which time the mice were randomly divided into 3 treatment groups: saline (vehicle), Taxotere^®^ (15 mg/kg), or o(LA)_8_-DTX (50%) (25 mg/kg-DTX equivalent). Mice were given the corresponding dose in 150 μL retro-orbital injections once-weekly for three weeks. Body weights were recorded thrice weekly. Two days following the last dose, mice were euthanized and whole blood, tumors, kidneys, liver, and heart were collected. Tissues were fixed in 4% paraformaldehyde, dehydrated in alcohol, cleared in Citrisolv, and infiltrated with paraffin. Sections were de-waxed in Citrisolv and rehydrated through graded ethanol. Immunohistochemistry (IHC) staining was used for analysis and quantitation of markers of cell proliferation (Ki67) and apoptosis (cleaved caspase-3). For antigen retrieval, the sections were boiled in Vector Citrate-based Unmasking Solution and cooled to room temperature. Slides were then incubated in 3% H_2_O_2_ to quench the reaction, followed by blocking and overnight incubation at 4 °C with a 1:200 dilution of Ki67 or cleaved caspase-3 primary antibody in 2.5% goat serum in PBS, a series of PBS + Tween washes, and an hour-long incubation at room temperature in a 1:1000 dilution of biotinylated goat anti-rabbit IgG secondary antibody in 2.5% goat serum in PBS. Slides were stained with the Vector Vectastain ABC Elite kit and incubated with DAB Peroxidase Substrate Kit for 5–10 min. DAB oxidation was halted by submerging the slides in water. Slides were counterstained in hematoxylin, dehydrated in graded ethanol and CitriSolv, then mounted using Permount mounting. After slides were stained, images were captured using a DMLB microscope (Leica Microsystems; Buffalo Grove, IL, USA) and MicroPublisher Color RTV-5.0 CCD Camera (QImaging; Surrey, BC, Canada), blinded, and analyzed in ImageJ version 1.53c (National Institutes of Health; Bethesda, MD, USA) via the Cell Counter and counted manually. Positively stained cells were counted as well as a total count.

For all histological stains, a Welch’s t-test was conducted to identify differences among means. Two-way ANOVA was used to compare body weight changes in tumor-bearing mice. A difference of *p* < 0.05 was considered significant. All statistics were performed using GraphPad statistical software version 9.2.0 (San Diego, CA, USA).

## 3. Results and Discussion

### 3.1. Synthesis and Characterization of o(LA)_8_-DTX

**Figure 2:**
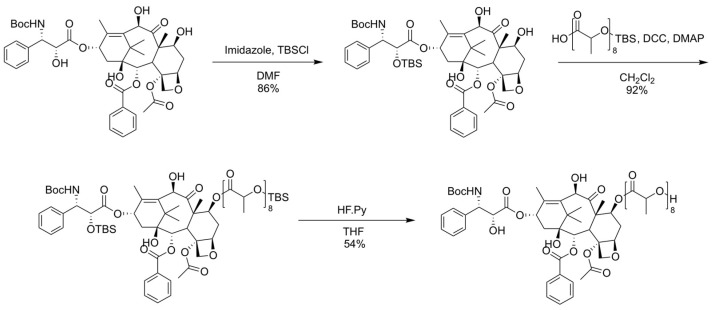
Synthetic route of o(LA)_8_-DTX prodrug.

The synthesis of the docetaxel prodrug was carried out according to a previously described protocol [26], and the scheme is depicted in Figure 2. We chose to modify DTX at the 7-OH position to preserve microtubule binding and biological activity [27]. Briefly, protection of the 2′-OH group of DTX was achieved using TBS, followed by conjugation of TBS-o(LA)_8_ on the 7-OH position via DCC/DMAP coupling. Following deprotection, o(LA)_8_-DTX prodrug was purified with CombiFlash (>95%) and dried in vacuo. ^1^H NMR and LCMS were used to confirm the structure (Figure S3) and purity (Figure S4) of o(LA)_8_-DTX, respectively.

### 3.2. Loading and Characterization of o(LA)_8_-DTX-Loaded PEG_4k_-b-PLA_2.2k_ Micelles

Loading of DTX or o(LA)_8_-DTX into PEG-*b*-PLA micelles was achieved using a thin film hydration method, and the micelle properties are displayed in Table 1. Two weight loadings were targeted, 10 and 50 wt% (hereby referred to as o(LA)_8_-DTX (10%) and o(LA)_8_-DTX (50%)), to assess the effect of prodrug loading on micelle properties. DTX micelles were only prepared at 10% loading due to the instability of this formulation. The z-average hydrodynamic diameters, as measured by DLS, ranged from 30–32 nm and 95–110 nm for o(LA)_8_-DTX (10%) and o(LA)_8_-DTX (50%), respectively. Due to the enhanced compatibility with the PLA core, nearly 100% encapsulation efficiency was achieved for both target weight loadings and 20 mg/mL o(LA)_8_-DTX (~12 mg/mL DTX-equivalent) could be solubilized in aqueous media. While similar loading efficiency could be achieved for unmodified DTX, o(LA)_8_-DTX proved to be more stable within the micelle core; no significant differences in prodrug content and particle size were observed after 3 months at 25 °C. This is in sharp contrast to DTX-loaded micelles, which precipitated out within 24 h.

### 3.3. In Vitro Drug or Prodrug Release from Micelles

As with many other nano-assemblies, Taxotere^®^ is a fast-releasing formulation. A recent in vitro drug release study showed that DTX release from Taxotere^®^ was complete after just 10 min [28]. To determine if we had successfully developed a delayed release formulation, we monitored the release of o(LA)_8_-DTX from PEG-*b*-PLA micelles using a dialysis method (Figure S5). Interestingly, the t_1/2_ of o(LA)_8_-DTX (50%) was higher than o(LA)_8_-DTX (10%): 39.3 and 16.1 h, respectively. These results contrast previous literature suggesting higher drug loading leads to faster drug release [29]. However, both of these formulations demonstrated a sustained release relative to DTX (10%) (t_1/2_ = 3.0 h). 

### 3.4. Backbiting Conversion of o(LA)_8_-DTX

**Figure 3:**
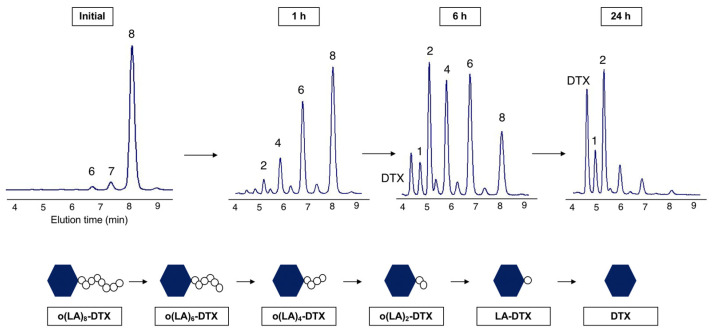
Representative RP-HPLC chromatograms of intramolecular backbiting of o(LA)_8_-DTX in 1:1 ACN:PBS. Numbered peak labels refer to o(LA)_n_ chain length. The elution time of o(LA)_8_-DTX is 8.1 min. As backbiting begins, shorter, even-numbered o(LA)_n_ chain length species with shorter elution times are formed, and DTX is gradually produced over time.

**Figure 4:**
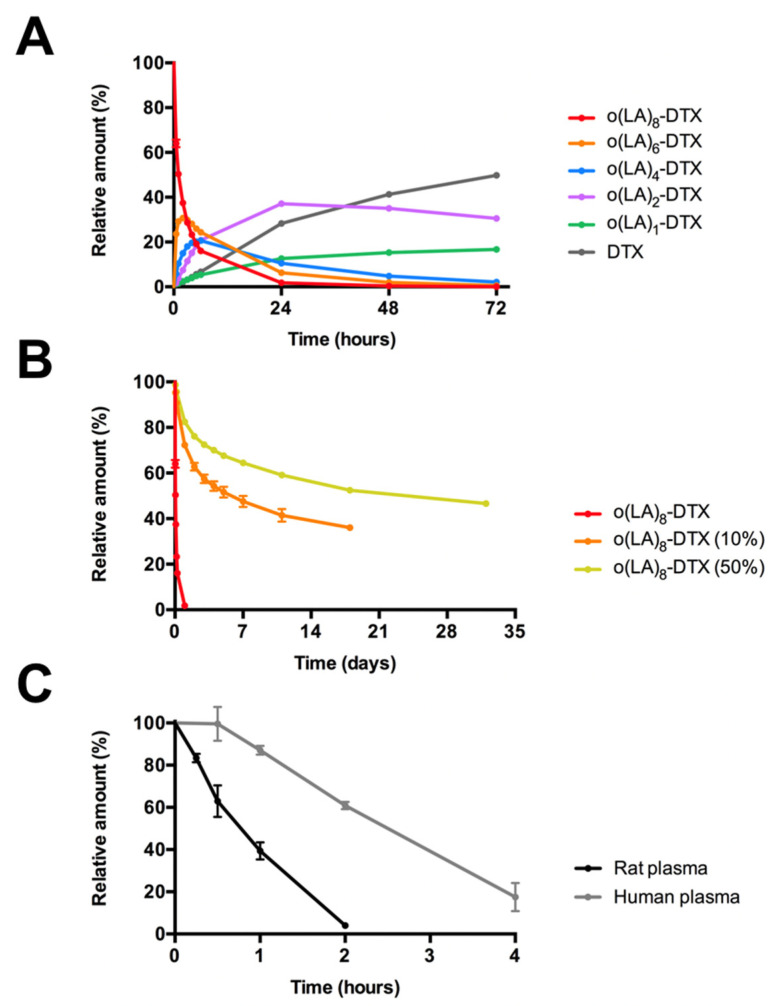
In vitro conversion and stability of o(LA)_8_-DTX at 37 °C (n = 3, mean ± SD). (**A**) Relative amounts of o(LA)_8_-DTX and each intermediate species after incubation in a 1:1 mixture of ACN and 10 mM PBS, pH 7.4 at 1 mg/mL. (**B**) Stability of o(LA)_8_-DTX in free form or in micelles at 1 mg/mL in PBS. (**C**) In vitro stability of o(LA)_8_-DTX incubated in rat or human plasma at a final concentration of 50 μM. Importantly, these results show that the structure of o(LA)_8_-DTX remains preserved within the micelle, but upon release, it is quickly converted to active DTX through backbiting or esterase-mediated degradation for drug action.

Previous studies have demonstrated that o(LA)_n_ containing a free hydroxyl end group is degraded via an intramolecular backbiting mechanism in which nucleophilic attack of the penultimate carbonyl group by the end hydroxy group cleaves two lactic acid units at a time [30]. This unique mechanism eliminates the reliance on enzymatic activity for complete prodrug conversion. We observed the backbiting conversion of unencapsulated o(LA)_8_-DTX in a 1:1 mixture of ACN and PBS (10 mM, pH 7.4) at 37 °C. Within 1 h of incubation, even-numbered degradation species, o(LA)_6_-, o(LA)_4_- and o(LA)_2_-DTX, were observed by RP-HPLC (Figure 3) and the t_1/2_ of o(LA)_8_-DTX was calculated as 6.3 h (Figure 4A). However, conversion from o(LA)_2_-DTX to DTX was slow, presumably due to steric hindrance, and only ~50% of o(LA)_8_-DTX was fully converted to DTX after 72 h. Previous work in our lab has shown that full conversion to the parent drug is not necessary for bioactivity [25], but further studies on the primary metabolites are needed to confirm this for o(LA)_8_-DTX. The low abundance of odd-numbered species suggested intramolecular backbiting, rather than random chain-end scission, was occurring.

In addition to free o(LA)_8_-DTX, the backbiting rate was also examined in o(LA)_8_-DTX-encapsulated micelles (10 and 50%) at 37 °C (Figure 4B and Figure S6). The nonpolar micelle core hindered the degradation of o(LA)_8_-DTX; t_1/2_ for o(LA)_8_-DTX (10%) and o(LA)_8_-DTX (50%) were 12.5 and 32.1 days, respectively. Moreover, full conversion to parent DTX was delayed. After three weeks, less than 10% of the prodrug had been fully converted to DTX at both weight loading levels.

### 3.5. In Vitro Plasma Stability of o(LA)_8_-DTX

To assess the stability of the prodrug in biologically-relevant media, o(LA)_8_-DTX was incubated in both rat and human plasma at 37 °C and monitored using RP-HPLC. As shown in Figure 4C, o(LA)_8_-DTX was rapidly hydrolyzed in both types of plasma, producing mainly o(LA)_2_-DTX, LA-DTX, and DTX, proving the o(LA)_8_ pro-moiety to be a good substrate for esterases. Although conversion was quick under both conditions, the half-life of o(LA)_8_-DTX in rat plasma was shorter (t_1/2_ = 0.77 h vs. 2.5 h in human plasma) due to the higher level of plasma esterases in rodents relative to humans [31].

### 3.6. In Vitro Cytotoxicity of o(LA)_8_-DTX

**Figure 5:**
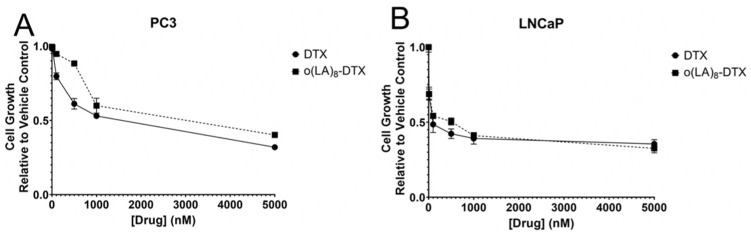
In vitro cytotoxicity of o(LA)_8_-DTX relative to parent drug DTX in (**A**) PC3 and (**B**) LNCaP prostate cancer cells. Cells were incubated with drug-treated media for 48 h (n = 3; mean ± standard error of the mean (SEM)).

The in vitro cytotoxicity of o(LA)_8_-DTX relative to DTX was assessed using the CellTiter Blue Viability Assay in 2 different human prostate cancer cell lines: PC3 and LNCaP (Figure 5). Cells were incubated with unencapsulated DTX or o(LA)_8_-DTX diluted in culture media for 48 h. Both DTX and o(LA)_8_-DTX proved to be more potent against the androgen-sensitive LNCaP cell line (IC_50_ = 84.9 and 302 nM, respectively), consistent with previous reports in which androgen receptor status of prostate cancer cells is related to DTX sensitivity [32]. While the antiproliferative effects were not as strong against PC3 cells, it was encouraging that the IC_50_ of o(LA)_8_-DTX (1.3 μM) was similar to that of unmodified DTX (1.2 μM).

### 3.7. In Vivo Antitumor Efficacy in Prostate CANCER Model

**Figure 6:**
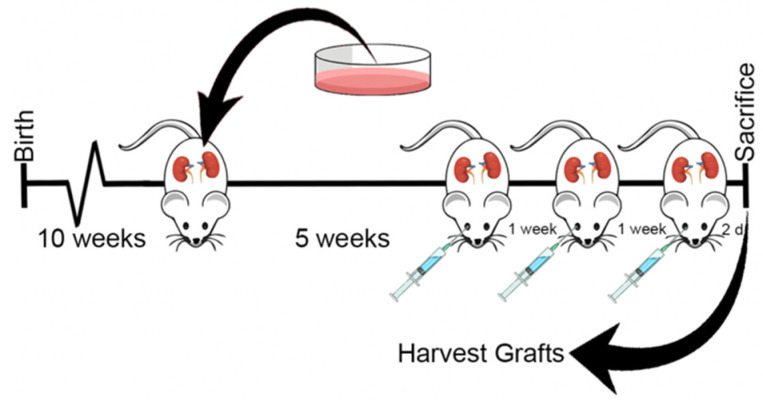
Treatment outline for PC3 and LNCaP tumor-bearing nude mice. Harvested PC3 and LNCaP prostate cancer cells were implanted under the kidney capsules of 10-week-old mice (one cell line on each kidney) and allowed to grow for five weeks. Mice were dosed retro-orbitally with vehicle (saline), Taxotere^®^ (15 mg/kg), or o(LA)_8_-DTX (50%) (25 mg/kg DTX-equivalent) once-weekly for three weeks. Two days following last treatment, mice were euthanized, and tumors were harvested for IHC analysis.

**Figure 7:**
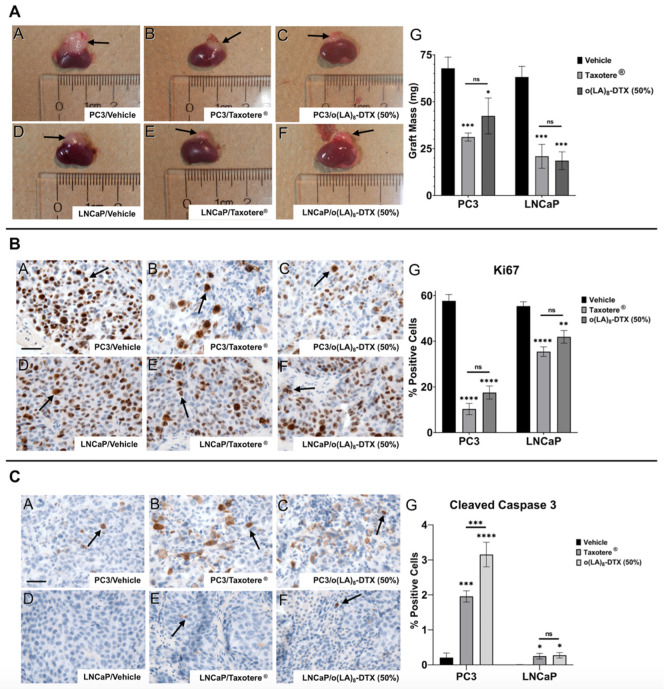
In vivo efficacy of o(LA)_8_-DTX-loaded micelles (n = 5 or 6; mean ± SEM). (**A**) Representative images of whole kidneys with grafted PC3 (A–C) or LNCaP (D–F) cells. Graft weights of resected tumors (G). (**B**) Representative IHC staining for Ki67, a proliferation marker, in PC3 (A–C) and LNCaP (D–F) tumors and the corresponding quantitation (G). (**C**) Representative IHC staining for cleaved caspase-3, a marker of apoptosis, in PC3 (A–C) and LNCaP (D–F) tumors and the corresponding quantitation (G). Representative scale bars in Panel A = 50 µm. * *p* < 0.05; ** *p* < 0.01; *** *p* < 0.001; **** *p* < 0.0001.

Given the promising results *in vitro*, both PC3 and LNCaP cell lines were chosen for the xenograft study in nude mice (Figure 6). Cells were implanted under the kidney capsules due to the high take rates, optimal vasculature, rich nutrient supply, and natural growth rates of tumors in this model [33]. Additionally, 2 different cell lines could be tested in one mouse at the same time. Taxotere^®^ was dosed at its weekly maximum tolerated dose (MTD) of 15 mg/kg [16], and we hypothesized that o(LA)_8_-DTX (50%) could be dose-escalated (25 mg/kg DTX-equivalent) due to the absence of Tween 80 and ethanol. Following three weekly injections, both Taxotere^®^ and o(LA)_8_-DTX (50%) significantly decreased tumor volume and mass relative to the vehicle control in both PC3 and LNCaP xenografts (Figure 7A). There were no statistically significant differences between treatment groups in either cell line. It is important to note that mice received only one cycle of treatment due to the potential for renal failure from tumor burden. We speculate that if more doses were administered, o(LA)_8_-DTX (50%) would prove to be more efficacious than Taxotere^®^ due to the improved carrier stability and prolonged DTX exposure, but further studies are needed to test this hypothesis.

Following euthanasia, tumors were resected and sectioned for IHC staining. In both PC3 and LNCaP tumors, IHC revealed a marked decrease in Ki67-positive cells, a marker of cell proliferation, for both treatment groups relative to the control (Figure 7B). While there was no significant difference in proliferation between Taxotere^®^ and o(LA)_8_-DTX (50%), o(LA)_8_-DTX (50%) induced more cell apoptosis than both Taxotere^®^- and vehicle-treated mice in the PC3 xenograft, as measured by cleaved caspase-3 (Figure 7C). Although there appeared to be an increase in apoptotic cells in Taxotere^®^ and o(LA)_8_-DTX (50%) mice, the low-to-negligent levels of cleaved caspase-3 in LNCaP tumors made quantitative analysis and comparisons challenging.

**Figure 8:**
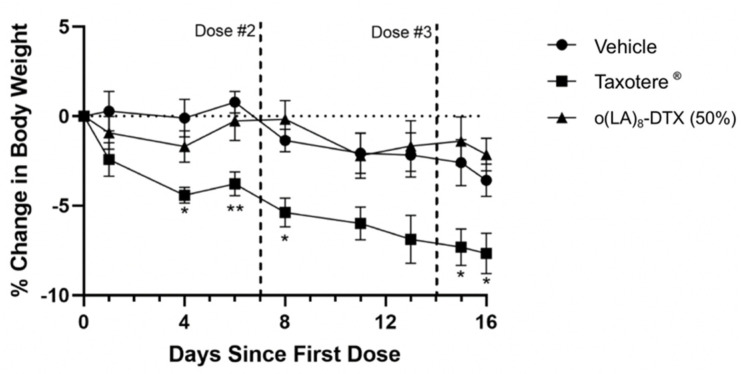
Body weights of PC3 and LNCaP tumor-bearing nude mice following first treatment with vehicle, Taxotere^®^, or o(LA)_8_-DTX (50%) (n = 5 or 6; mean ± SEM). While Taxotere^®^ mice lost a significant amount of weight relative to the control group, there were no significant differences between vehicle- and o(LA)_8_-DTX (50%)-treated mice. * *p* < 0.05; ** *p* < 0.01.

The toxicity profile of chemotherapy formulations is also of importance in addition to anticancer efficacy. As shown in Figure 8, mice treated with Taxotere^®^ experienced weight loss soon after their first dose and at the conclusion of the experiment had an average of 8% body weight decrease. In contrast, the decrease in body weights of o(LA)_8_-DTX (50%)-treated mice was minimal and comparable to those treated with vehicle, despite the increase in dose relative to Taxotere^®^. Since o(LA)_8_-DTX (50%) was well tolerated, it can be hypothesized that the formulation could be further dose-escalated, but additional studies establishing the MTD are needed.

## 4. Conclusions

In summary, we have synthesized a novel ester prodrug of DTX (o(LA)_8_-DTX) for loading in PEG-*b*-PLA micelles at 50% *w*/*w* prodrug loading. Addition of the o(LA)_8_ pro-moiety led to a significant improvement in micelle stability and a more sustained payload release relative to unmodified DTX. While o(LA)_8_-DTX remained intact within the micelle, it was converted to DTX by intramolecular backbiting and plasma esterase-mediated degradation following its release. In a murine xenograft model, o(LA)_8_-DTX (50%) proved to be as efficacious as Taxotere^®^ against both PC3 and LNCaP human prostate cancer tumors, with less toxicity. These results provide justification for further preclinical evaluation of o(LA)_8_-DTX with the goal of improving treatment outcomes for prostate cancer patients.

## Figures and Tables

**Table 1:** Physicochemical properties of DTX- or o(LA)_8_-DTX-loaded PEG-*b*-PLA micelles prepared by thin film hydration method (n = 3; mean ± standard deviation (SD)).

Drug/Prodrug (Target wt Loading %)	Actual wt Loading (%)	Encapsulation Efficiency (%)	Z-Average Particle Size (nm)	PDI	Room Temperature Stability	Release t_1/2_ (h)
DTX (10%)	9.7 ± 0.1	97.2 ± 2.5	30.6 ± 1.7	0.18 ± 0.03	<24 h	3.0
o(LA)_8_-DTX (10%)	9.8 ± 0.6	98.2 ± 5.9	31.2 ± 0.5	0.07 ± 0.02	>3 months	16.1
o(LA)_8_-DTX (50%)	49.0 ± 0.8	98.1 ± 1.7	103.4 ± 10.9	0.10 ± 0.02	>3 months	39.3

## Data Availability

Not applicable.

## Supplementary Materials

The following are available online at https://www.mdpi.com/article/10.3390/nano11102745/s1. Figure S1: ^1^H NMR spectrum of 2′-TBS-DTX. Figure S2: ^1^H NMR spectrum of 2′-TBS-DTX-o(LA)_8_-TBS. Figure S3: ^1^H NMR spectrum of o(LA)_8_-DTX. Figure S4: Purity of o(LA)_8_-DTX and calculated molecular weight using LCMS. Figure S5: In vitro release of DTX or o(LA)_8_-DTX from PEG-*b*-PLA micelles. Figure S6: Representative RP-HPLC chromatograms of intramolecular backbiting of o(LA)_8_-DTX loaded in PEG-*b*-PLA micelles.
