# Supplementary material for: Oligo(Lactic Acid)8-Docetaxel Prodrug-Loaded PEG-b-PLA Micelles for Prostate Cancer
Source: Nanomaterials (Basel). 2021 Oct 17;11(10):2745. doi: 10.3390/nano11102745 (PMC8540550; doi:10.3390/nano11102745)
Supplement: Supplementary file 1 [file nanomaterials-11-02745-s001.zip › nanomaterials-1380985-SI.pdf]

Oligo(lactic acid)$_8$-Docetaxel Prodrug-Loaded PEG-*b*-PLA Micelles for Prostate Cancer

Lauren Repp [1], Christopher J. Unterberger [1], Zhengqing Ye [2], John B. Feltenberger [2], Steven M. Swanson [1], Paul C. Marker [1] and Glen S. Kwon [1,*]

[1] Pharmaceutical Sciences Division, School of Pharmacy, University of Wisconsin-Madison, Madison, WI 53705, USA; lrepp@wisc.edu (L.R.); cunterberger@wisc.edu (C.J.U.); steve.swanson@wisc.edu (S.M.S.); paul.marker@wisc.edu (P.C.M.)

[2] Medicinal Chemistry Center, School of Pharmacy, University of Wisconsin-Madison, Madison, WI 53705, USA; zhengqing.ye@wisc.edu (Z.Y.); john.feltenberger@wisc.edu (J.B.F.)

* Correspondence: glen.kwon@wisc.edu

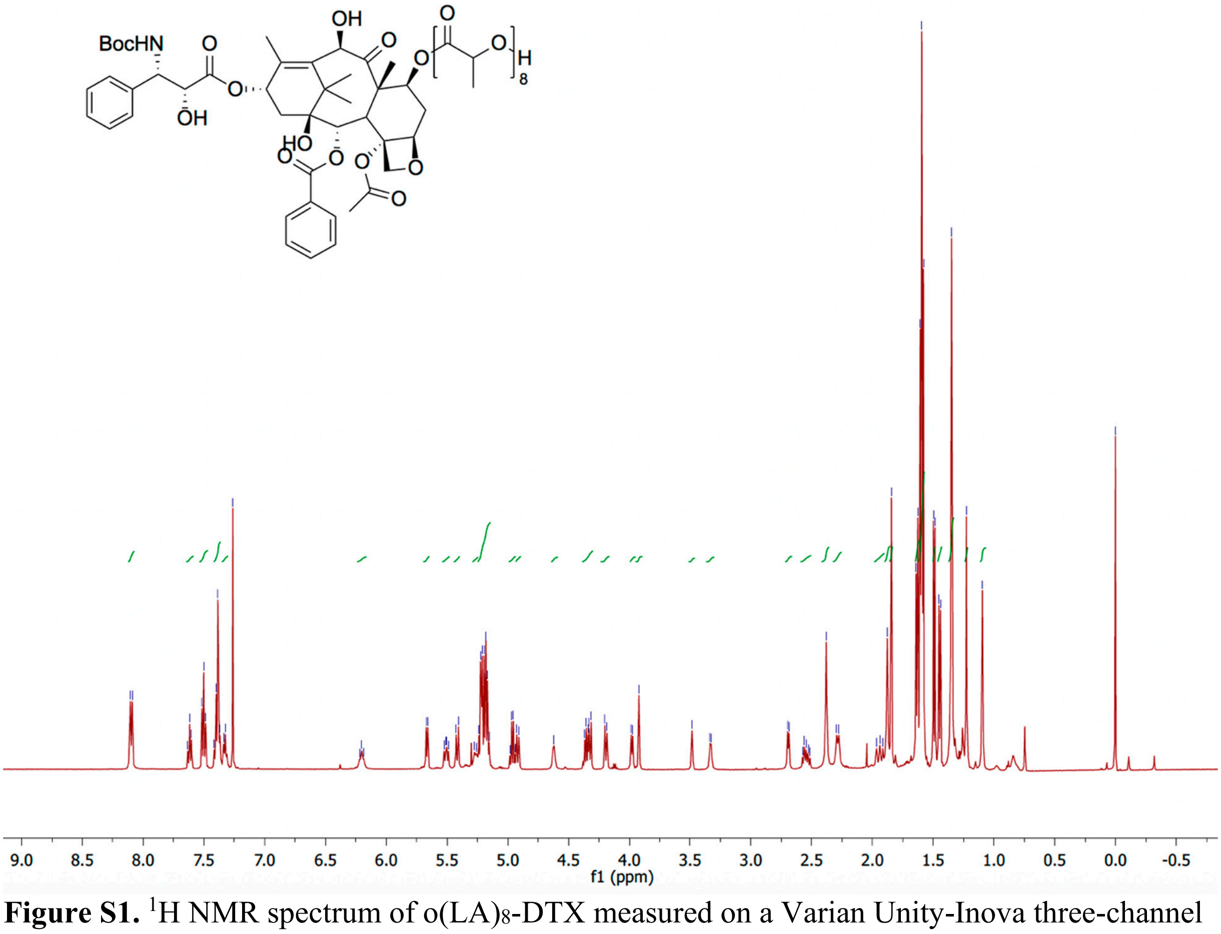

Figure S1. ^{1}H NMR spectrum of o(LA)$_8$-DTX measured on a Varian Unity-Inova three-channel 500 MHz NMR spectrometer.

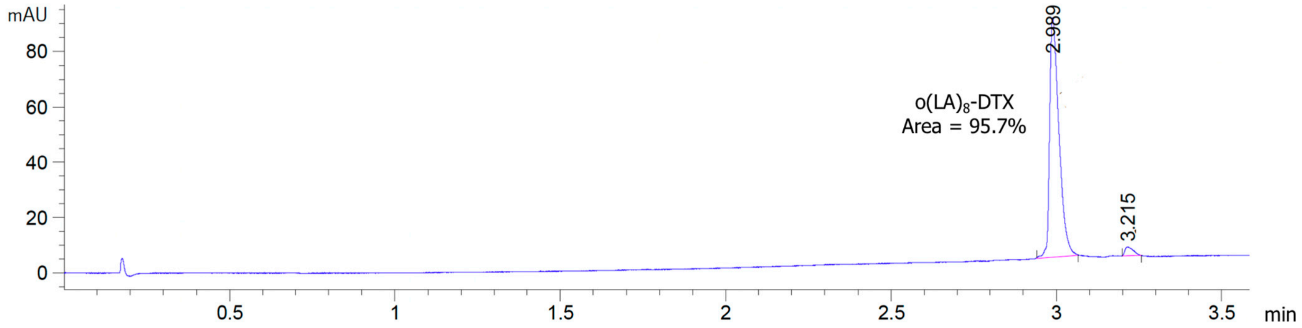

Molecular weight calculated $[M+NH_4]^+$	1401.5
Molecular weight found $[M+NH_4]^+$	1401.9

Figure S2. Purity of $o(LA)_8$-DTX and calculated molecular weight of corresponding peak using LCMS.

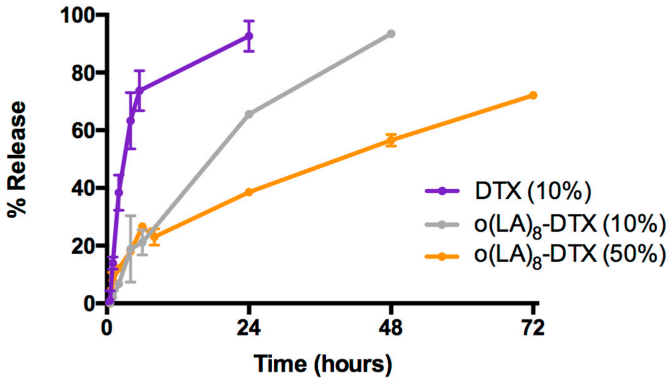

Figure S3. *In vitro* release of DTX or o(LA)8-DTX from PEG-*b*-PLA micelles in PBS (10 mM, pH 7.4) at 37 °C (n = 3; mean ± SD).

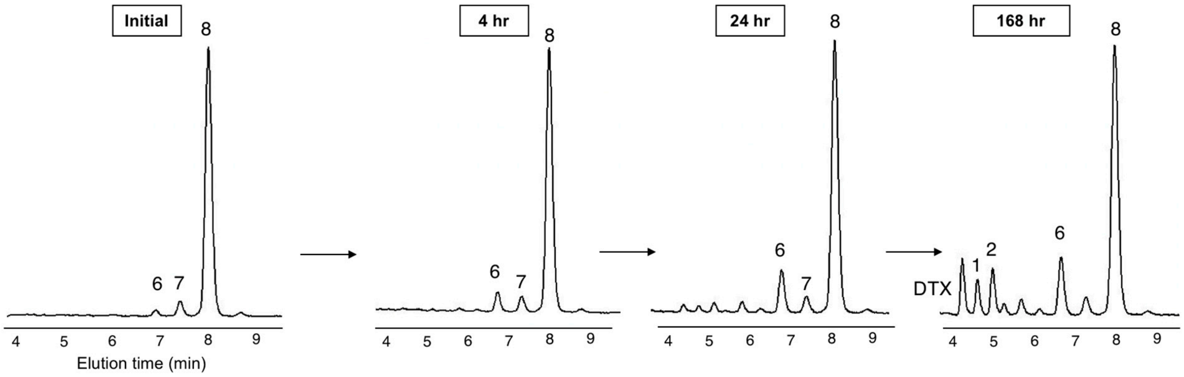

Figure S4. Representative RP-HPLC chromatograms of intramolecular backbiting of o(LA)$_8$-DTX loaded in PEG-*b*-PLA micelles at 37 °C. Degradation of o(LA)$_8$-DTX is significantly slower in the nonpolar environment of the micelle core.

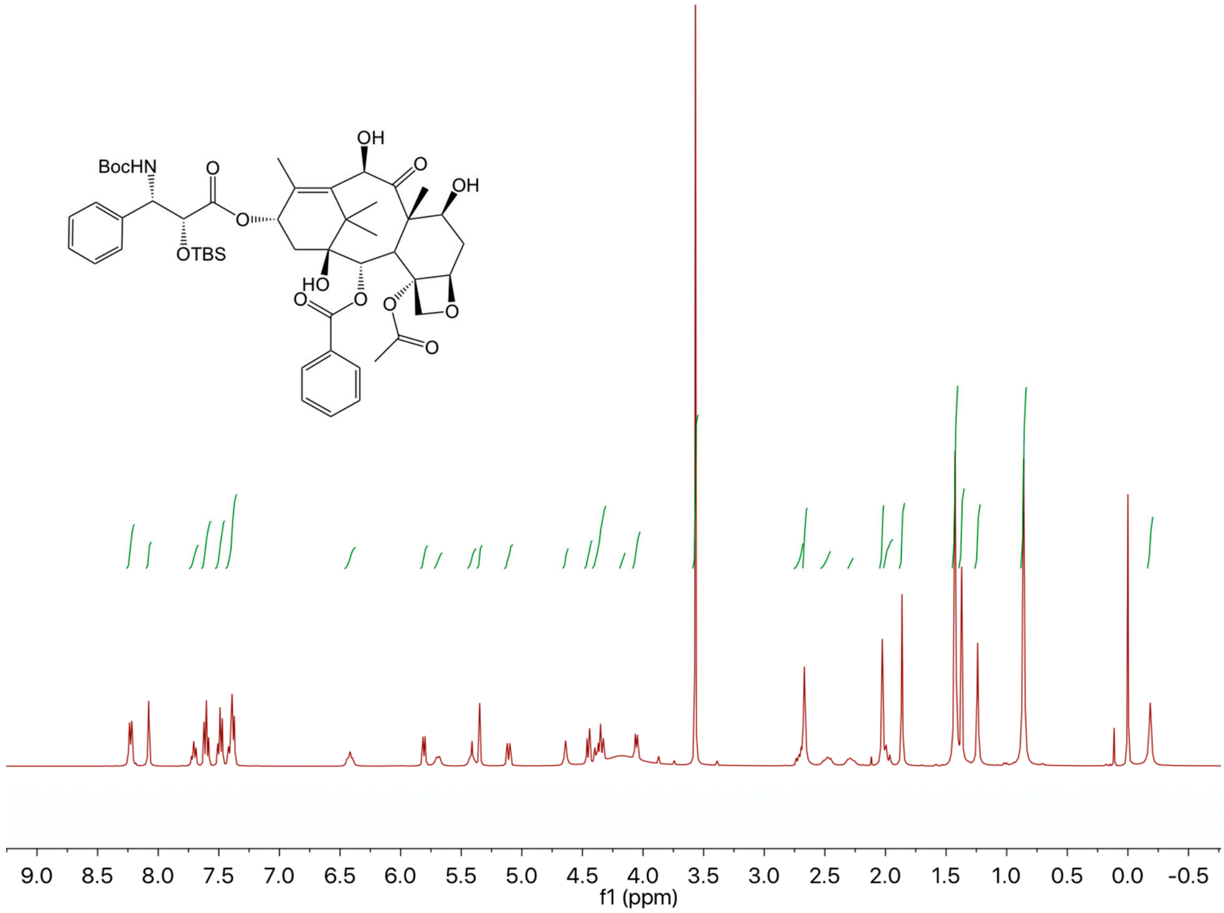

Figure S5. ^{1}H NMR spectrum of 2’-TBS-DTX.

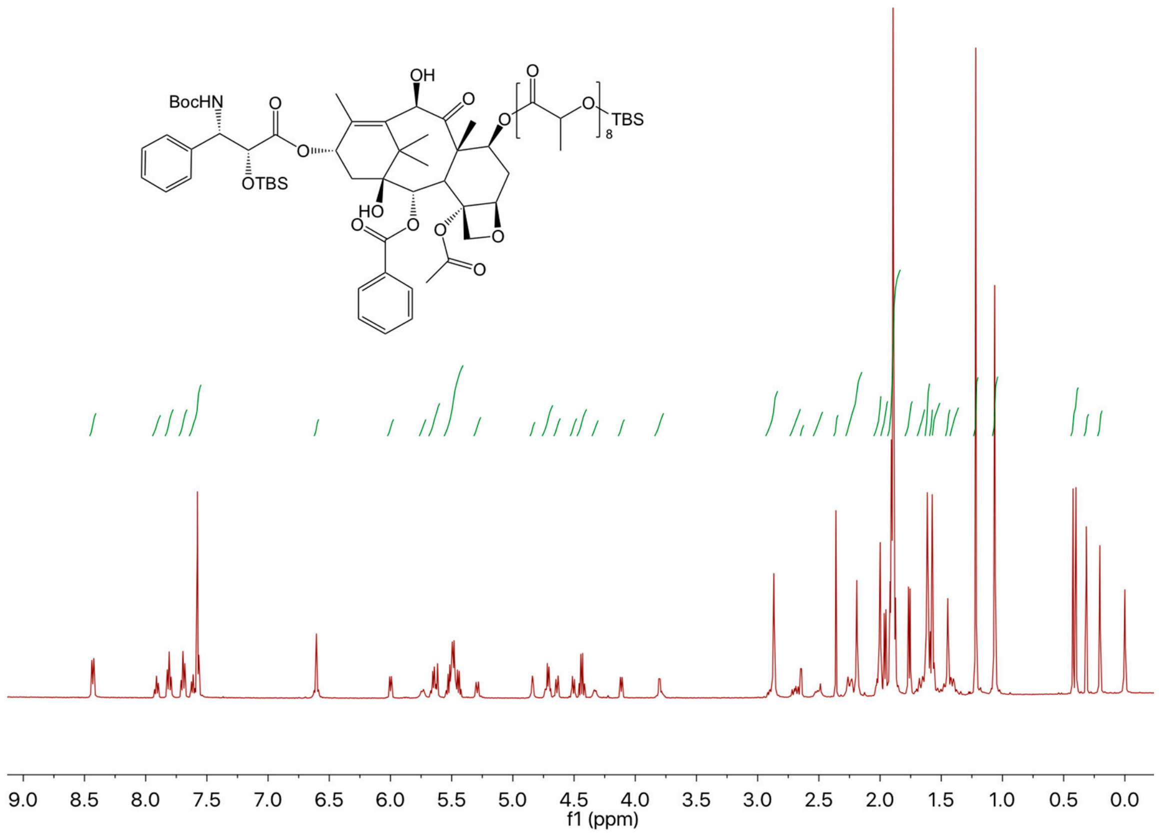

Figure S6. ^{1}H NMR spectrum of 2'-TBS-DTX-o(LA)$_8$-TBS.
